# Suppression of AURKA alleviates p27 inhibition on Bax cleavage and induces more intensive apoptosis in gastric cancer

**DOI:** 10.1038/s41419-018-0823-3

**Published:** 2018-07-16

**Authors:** Daisen Hou, Zhihui Che, Ping Chen, Wenli Zhang, Yiwei Chu, Dongqin Yang, Jie Liu

**Affiliations:** 1 Department of Digestive Diseases of Huashan Hospital and Institute of Biomedical Sciences, Fudan University, Shanghai, China; 2 Department of Digestive Diseases, National Clinical Research Center for Aging and Medicine, Huashan Hospital, Fudan University, Shanghai, China; 3 Institute of Biomedical Sciences and Department of Immunology, School of Basic Medical Sciences, Fudan University, Shanghai, China

## Abstract

Bax is a key molecule in mitochondria-apoptosis pathway, however it is not always an efficient apoptosis inducer in chemotherapeutic agents-treated cancer cells. Here, we found that specific inhibition of AURKA by MLN8237-induced calpain-mediated Bax cleavage at N-terminal 33th asparagine (c-Bax) to promote apoptosis. The c-Bax, as Bax, could also efficiently located to mitochondria but c-Bax is a stronger apoptosis inducer than Bax. Morever, c-Bax-induced apoptosis could not be blocked by the canonical Bax inhibitor, Bcl-2. Further study found p27 was degraded and subsequently Bax was transformed to c-Bax through calpain. Also, p27 efficiently inhibited Bax cleavage and p27 knockdown sensitized apoptosis through Bax cleavage when cancer cells were treated with MLN8237. It is also demonstrated that the anti-apoptotic role of p27 lies its cytoplasmic localization. Finally, we found that the positive correlation between AURKA and p27 in advanced gastric cancer patients. In conclusion, we found that MNL8237 suppressed cell growth by regulating calpain-dependent Bax cleavage and p27 dysregulation in gastric cancer cells.

## Introduction

Gastric cancer (GC) is a heavy burden to public health as its overall mortality ranked third in cancer-related deaths worldwide in 2012^1^. The majority of patients suffering from gastric cancer are diagnosed at the advanced stages accompanied with malignant proliferation, dysfunction of cell cycle, and distant metastasis. Currently, the therapeutic targets and therapies for gastric cancer are limited. Therefore, the molecular mechanisms accounting for the initiation and progression of GC need to be investigated to better figure out the way to cure GC.

Cyclin-dependent kinase inhibitor 1B (p27) from the Cip/Kip family is a well-known cancer suppressor that interacts with CDK 4/6-Cyclin D or CDK2-Cyclin E/A complex to control cell cycle^2^. Numerous pathological studies have verified p27 downregulation in various type of tumors, including breast^3^, colon^4^, lung^5^, liver^6^, and stomach^7^. p27 is also dysregulated in gastric cancer and is associated with advanced stage and invasiveness of gastric cancer^8^. The CDK-inhibitory activity of p27 is controlled by the concentration, subcellular localization and phosphorylation status. Although p27 is not a classic tumor cancer suppressor like p53; as it is rarely mutated or deleted in human cancers^9^, it is frequently deregulated in cancer-p27 protein levels are reduced or the protein is mislocalized in most cancers and this is associated with a poor prognosis^10^.

Aurora kinases, including Aurora A, B, and C, are serine/threonine kinases with major roles in mitosis and cytokinesis. At the start of S phase during mitosis, Aurora A (AURKA) is found at centrosomes and is essential for centrosome maturation, spindle assembly, and orientation. Aurora B (AURKB) localizes to chromosomes and spindle to construct the chromosomal passenger complex (CPC) to control chromosome condensation orientation and cytokinesis execution. Compared with Aurora B inhibition, AURKA inhibition has shown promising clinical results in several clinical trials.

MLN8237(Alisertib) is a selective AURKA inhibitor. Cancer cells treated with MLN8237 showed mitotic arrest and polyploidy, senescence and apoptosis^11^. In mouse tumor models of neuroblastoma^12^ and lymphoma^13^, MLN8237 treatment resulted in tumor regression and survival rate increase. Importantly, AURKA inhibition by MLN8237 triggered MYC degradation and tumor regression in a MYCN-driven mouse model of neuroblastoma^14^.

We used MLN8237 to investigate the functions of AURKA in apoptosis. Knockdown of p27 by siRNA in gastric cancer cells increased MLN8237-induced Bax cleavage and localization to mitochondrial to induce the mitochondrial apoptosis pathway. The cancer-promoting function of p27 may be related to its location in the cytoplasm rather than the nucleus. Further experiments showed that p27 and Bax were both calpain substrates. Once calpain was activated, p27 degradation and Bax cleavage occurred subsequently. We also found c-Bax-induced apoptosis could not be blocked by the canonical anti-apoptosis protein, Bcl-2. Finally, both AURKA and p27 were overexpressed in gastric cancer tissues. Thus, our study provides the first piece of appealing evidence supporting the notion that AURKA is an attractive target for the development of gastric cancer therapeutics.

## Results

### Gastric cancer cell growth was suppressed by AURKA inhibition upon MLN8237 treatment

**Fig. 1:**
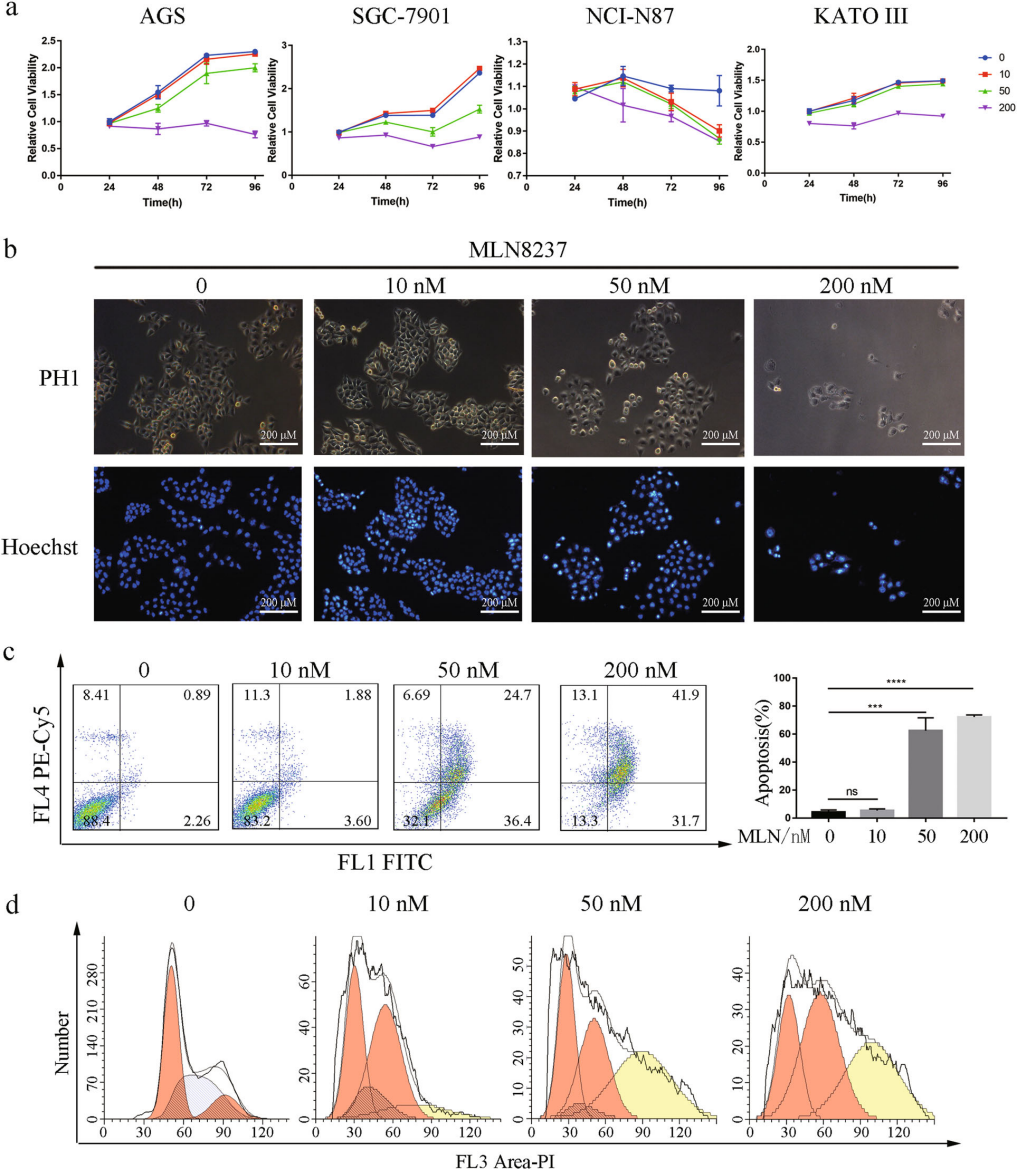
Cell cytotoxicity of gastric cancer cells induced by MLN8237 treatment. **a**, **b** MLN8237 suppressed the proliferation of gastric cancer cells. AGS, SGC-7901, NCI-N87 and KATO III cells were treated with MLN8237 at the indicated concentrations for 24–96 h, then counted cell number **a** with MTS assay or **b** with Hoechst 33342 staining in AGS cell line. The mean and SDs of the plots were obtained from three wells within three independent assays. **c** MLN8237 treatment induced apoptosis of AGS cell line as determined by Annexin V/propidium iodide double staining and FCM. AGS was treatment with MLN8237 for 72 h. Statistical analysis from triplicates of different biological experiments. **d** MLN8237 treatment induced G2/M arrest and polyploidy in gastric cancer cells. The cell cycle profile was determined by treating AGS cells with the indicated concentration of MLN8237 for 72 h, staining the cells with propidium iodide staining and analyzing by FCM. This staining is representative of three different independent experiments. This immunoblot is representative of three independent experiments. Asterisk (*) indicates a significant difference. ***P < 0.001, ****P < 0.001, two-tailed Student’s *t* test

MLN8237 is a selective AURKA inhibitor which is a potential therapeutic agent for B-cell and T-cell non-Hodgkin lymphoma, breast, lung and prostate tumors, neuroblastoma and multiple myeloma^15^. However, its effect on gastric cancer is still unknown. We determined the efficiency of MLN8237 treatment in gastric cancer cells, including AGS, SGC-7901, NCI-N87, and KATO III. MTS colorimetric assay and Hoechst 33342 staining showed that MLN8237 had a dose- and time-dependent inhibition manner on all tested gastric cancer cell lines (Fig. 1a, b). Annexin V/propidium iodide staining showed that 50 nM and 200 nM MLN8237 for 72 h significantly induced apoptosis (Fig. 1c). Further, the cytotoxicity of MLN8237 was verified in HepG2 and BEL-7404 hepatocarcinoma cell lines (Figure S1). The specific silencing of AURKA in AGS also inhibited cell proliferation and induced apoptosis (Figure S1c, S1d). Moreover, G2/M arrest was induced by MLN8237 in a time- and dose-dependent manner and MLN8237 treatment transformed cells from diploids to tetraploids (Fig. 1d, Figure S2a). However, MLN8237 treatment had no obvious effect in LO2 and MIHA liver normal cell lines (Figure S1b). These results confirm the impaired proliferation of gastric cancer cells by MLN8237.

### Bax and p27 were both cleaved by activating calpain pathway upon MLN8237 treatment

**Fig. 2:**
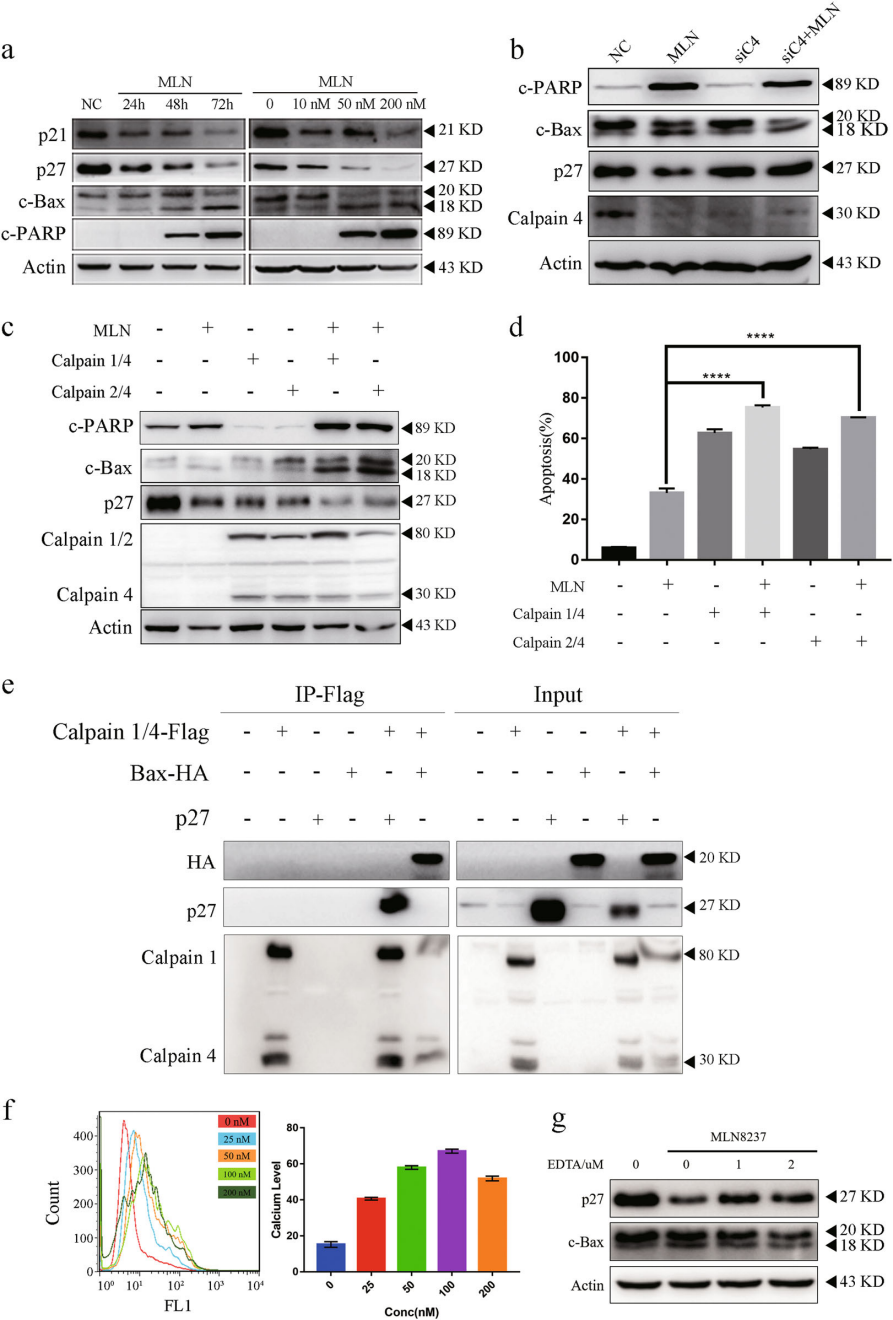
MLN8237 treatment activated the calpain pathway and induced apoptosis through p27 degradation and Bax cleavage in AGS cell line. **a** MLN8237 treatment led to p27 downregulation and Bax cleavage. AGS cells were cultured at the indicated concentration of MLN8237 for 72 h and incubated time at 200 nM. The protein samples were subjected to an immunoblotting analysis of p21, p27, c-Bax, c-PARP, and Actin. **b** Calpain knockdown attenuated the p27 turnover and Bax cleavage induced by the treatment of the cells with 200 nM MLN8237 for 72 h. Calpain 4 was knocked down and treated with 200 nM MLN8237 for 72 h in AGS. Protein expression was determined by immunoblotting analysis. **c** Immunoblotting analysis demonstrated that both calpain 1 and calpain 2 degraded p27 and cleaved Bax. Calpain 1/4 or calpain 2/4 were overexpressed in AGS gastric cancer cells and then treated with MLN8237 at 200 nM, and subjected to immunoblotting analysis for indicated protein expression. **d** Calpain 1 or calpain 2 enhanced apoptosis of MLN8237-treated AGS gastric cancer cells. Calpain 1/4 or calpain 2/4 were overexpressed and treated with 200 nM MLN8237 for 72 h. Apoptosis was determined by FCM. The experiment was performed independently three times. **e** Both p27 and Bax combined with calpain. Calpain 1/4-Flag was overexpressed with pCMV-p27 or Bax-HA plasmids for 24 h in 293 T. Equivalent cell lysate was immunoprecipitated with Flag beads and immunoblotted p27 and HA protein levels. **f**, **g** MLN8237-induced Ca^2+^ release to the cytoplasm. AGS was treated with MLN8237 for 72 h at indicated concentration and loaded with 5 μM Fluo-3/AM for 30 min at 37 °C. The labeled cells were analysis by FCM (**f**). **g** AGS was pre-incubated with 1 and 2 uM EDTA for 2 h and followed treated with 200 nM MLN8237 for 72 h. Error bars represent SD from three independent experiments. Asterisk (*) indicates a significant difference. **P < 0.01, ***P < 0.001, ****P < 0.001, two-tailed Student’s *t* test

We tested the effect of MLN8237 treatment on the apoptosis pathway and cell cycle pathway in AGS gastric cancer cell line. We found that c-PARP, which served as a marker of apoptotic cells, was upregulated. Meanwhile, p21 and p27, CDK–Cyclin complex inhibitors, were both downregulated (Fig. 2a). Bax is an important mitochondrial apoptosis activator that can form a heterodimer with Bcl-2 to suppress the apoptosis-inducing function. Surprisingly, we found Bax was cleaved when AGS was treated with MLN8237 (Fig. 2a). As p27 was downregulated, Bax cleavage was increased. As shown in Figure S3a, Bax cleavage and p27 degradation is only found in SMMC-7901 and HepG2. To confirm Bax cleavage was AURKA-dependent, we knocked down AURKA using RNAi and found that AURKA knockdown-induced Bax cleavage (Figure S4a). AURKA knockdown also enhanced MLN8237-dependent p27 downregulation and Bax cleavage (Figure S4b). Previous studies have demonstrated Bax could be cleaved by calpain^16^. To determine whether MLN8237-induced Bax cleavage was calpain-dependent, we utilized siRNA against the common regulatory subunit of calpain 1 and calpain 2 complexes (calpain 4), to inhibit calpain function. As shown in Fig. 2b, c-Bax (cleaved Bax) expression in the combined treatment group was significantly suppressed compared with that of the MLN8237 only-treatment group. AURKA knockdown-induced apoptosis was attenuated when calpain was knocked down (Figure S4c, d). Bax is a crucial apoptosis inducer in exogenous and endogenous stimulation. Bax knockdown significantly suppress apoptosis induced by MLN8237 with or without calpain overexpression, whinch indicated that the important role of Bax in mediating these processed. Calpain ovexpression induced increased apoptosis whereas Bax knockdown had no role in preventing this process, which suggested that other pathways exist (Figure S5a–c).

Interestingly, p27 had an opposite antagonistic effect to c-Bax (Fig. 2a). As a tumor suppressor, p27 can be cleaved and degraded by calpain^17^. These results suggested that both p27 and Bax were modified by calpain. To validate our conception, different isoforms of calpain were overexpressed in AGS. The expression of c-Bax was much higher in the MLN8237 group with calpain 1/2, calpain 4, and MLN8237 compared to the group treated with MLN8237 alone in AGS. Also, the overexpression of calpain caused a further downregulation of p27. However, there was no difference in the expression levels of p27 and c-Bax between calpain 1 and calpain 2 overexpression group, which indicates they shared similar function in degrading p27 and cleaving Bax (Fig. 2c). Skp2, the canonical E3 ligase of p27, had no effect on p27 downregulation and Bax cleavage upon MLN8237 treatment (Figure S6a). Activated caspase shared some similar function with calpain. However, the pan-caspase inhibitor Z-VAD-FMK did not have an effect on MLN8237-induced p27 degradation and Bax cleavage (Figure S6b). These results showed that calpain degraded p27 and transformed Bax into c-Bax subsequently. Approximately 33% of the cells treated with 25 nM MLN8237 were apoptotic. To our surprise, the combination of MLN8237 treatment and calpain overexpression strongly induced apoptosis to 75.3% and 70.3% in calpain 1 or calpain 2 overexpressed groups, respectively (Fig. 2d). To investigate further whether p27 degradation and Bax cleavage were indeed related to calpain, we performed combined immunoprecipitation/western blot experiments (Fig. 2e). As expected, an interaction between calpain and p27 or Bax was found. These results indicated that both p27 and Bax were regulated by calpain subsequently.

The archetypical members of calpain family, μ-calpain and m-calpain, were named for the concentration of calcium ions required for their activity^18^. We detected calcium concentrations using Fluo-3 AM calcium indicator and flow cytometry. As the MLN8237 concentration gradually increased, the calcium concentration gradually increased (Fig. 2f). Calcium signaling was detected by Fluorescence microscope in vitro (Figure S7a). In order to further investigate the Calcium Flux, AGS was previously incubated with calcium chelator EDTA for 2 h followed by MLN8237 treatment at 200 nM for 72 h. As shown in Fig. 2f, MLN8237-mediated Bax cleavage was inhibited by 1 and 2 uM EDTA. These results indicate that MLN8237 induces calcium signaling and activates calpain pathway.

### p27 silencing enhanced MLN8237-induced Bax cleavage and apoptosis in gastric cancer cells

**Fig. 3:**
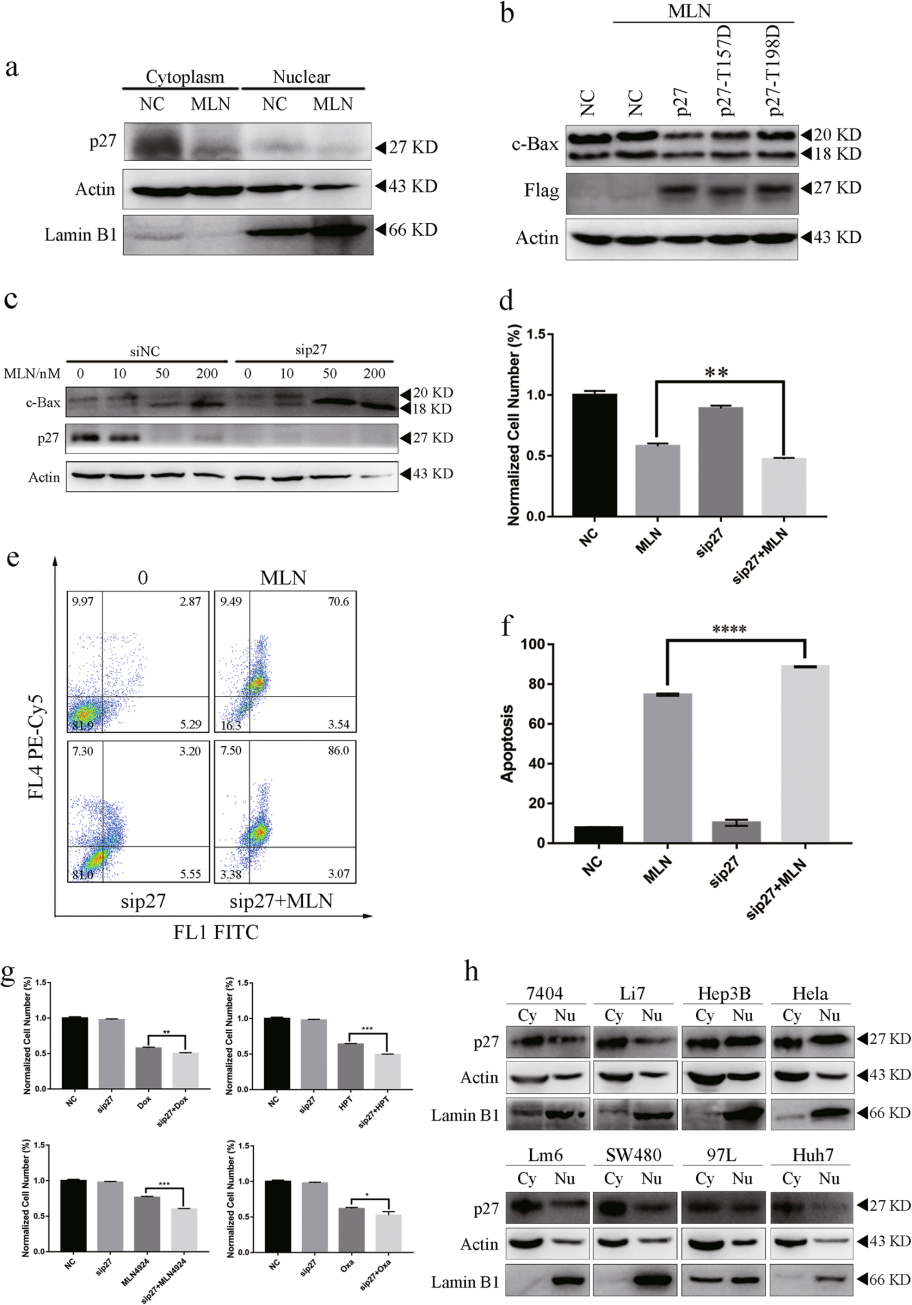
The protective role of cytoplasmic p27 in MLN8237-induced Bax cleavage and apoptosis in AGS gastric cancer cell line. **a** p27 distribution in the cytoplasm of AGS. Immunoblotting was subjected to detect p27 distribution after nuclear isolation according to manufacturer description. **b** p27 and phosphomimetic p27 mutant (T157D or T198D) overexpression inhibited MLN8237-induced Bax cleavage. AGS cells were transfected with Flag-tagged p27 or p27 mutant (T157D or T198D) and then treated with 200 nM MLN8237 for 72 h. **c**–**f** The combination of MLN8237 treatment and p27 silencing increased Bax cleavage. AGS cells were subjected to immunoblotting analysis **c** to determine the expression of the indicated proteins. MTS **d** was utilized to detect cell proliferation or FCM **e**, **f** to detect apoptosis level. 200 nM MLN8237 was incubated for 72 h in AGS. The mean and SDs of the plots were obtained from three wells within three independent MTS and apoptosis detection assays. The apoptosis staining experiment was performed independently three times. **g** Enhanced cytotoxicity of some chemotherapeutic agents after p27 silencing. 1 μM doxorubicin (Dox), 1 μM Hydroxycamptothecin (HPT), 0.3 μM MLN4924 or 10 μM oxaliplatin (Oxa) was added to culture after p27 silencing and subjected to MTS assay and immunoblotting at 72 h in AGS cell line. Error bars represent SD from three independent experiments. **h** The cytoplasmic localization of p27 in selected cancer cells. Immunoblotting was subjected to detect p27 distribution after nuclear isolation according manufacturer description. This immunoblot is representative of three independent experiments. Asterisk (*) indicates a significant difference. *P < 0.05, **P < 0.01, ***P < 0.001, ****P < 0.001, two-tailed Student’s t test

Our results showed that p27 antagonize Bax cleavage because of their inverse correlation upon MLN8237 treatment. As a cell cycle inhibitor and tumor suppressor, cytoplasmic p27 has functions that are distinct from its regulatory nuclear cell cycle inhibition functions. Therefore, we speculated that p27 had a protective role in MLN8237-treated gastric cancer cells. The nuclear extract assay showed that p27 was mainly mislocalized to cytoplasm in gastric cancer cells and that MLN8237 treatment induced downregulation of p27 levels in the cytoplasm (Fig. 3a). By introducing phosphomimetic p27 mutant (T157D or T198D) makes the p27 to be localized in the cytoplasm, and our results illustrated that overexpression of p27 or p27 mutant attenuates Bax cleavage upon MLN8237 treatment (Fig. 3b).

To elucidate the function of p27 in the MLN8237-induced cell proliferation arrest, we knocked down p27 using specific targeted siRNA in gastric cancer cells and found that p27 knockdown significantly increased MLN8237-induced Bax cleavage (Fig. 3c). Compared with the MLN8237 only group, the cell viability in the MLN8237-treated and p27 knocked down group was significantly reduced (Fig. 3d). Similarly, compared with the MLN8237-treated group, the percentage of apoptotic cells in the MLN8237-treated and p27 knocked down group increased by 14.9%, as measured using the Annexin V/PI double staining assay (Fig. 3e, f). Calpain promotes p38/MAPK phosphorylation and suppress apoptosis after stimulus^19^.

To further detail the protective role of p27 in AGS, we investigated the cytotoxicity of some chemotherapeutic agents in p27 knockdown gastric cancer cells. We found p27 knockdown enhanced cytotoxicity of tested chemotherapeutic agents (Fig. 3g). p27 cytoplasmic mislocalization may promote it from CDK inhibitor protein to protective protein in many cancer cells (Fig. 3h). Unlike the knockdown of p27, p21 knockdown opposed MLN8237-induced cell cytotoxicity and could not facilitate Bax cleavage (Figure S8a, b). Thus, cytoplasm p27, rather than nuclear p27, had a protective role in MLN8237-treated gastric cancer cell.

On the other hand, p38/MAPK mediate calpain activation, without affecting its phosphorylation^19^. Therefore, we investigated whether p38/MAPK was activated upon MLN8237 treatment. MLN8237 treatment caused the downregulation of p-p38 (T180/Y182) (Figure S9a). Suppression of p-p38 enhanced the inhibitory effect of MLN8237 on proliferation and increased apoptosis (Figure S9b-d). Meanwhile, p38/MAPK inhibition of SB203580 increased the sensitivity of the cells to MLN8237-induced Bax cleavage (Figure S9e). These results showed that MLN8237 and SB203580 had a higher cytotoxicity in tumor cells than either one used alone.

### c-Bax localizes to mitochondrial and induce cytochrome c release and apoptosis

**Fig. 4:**
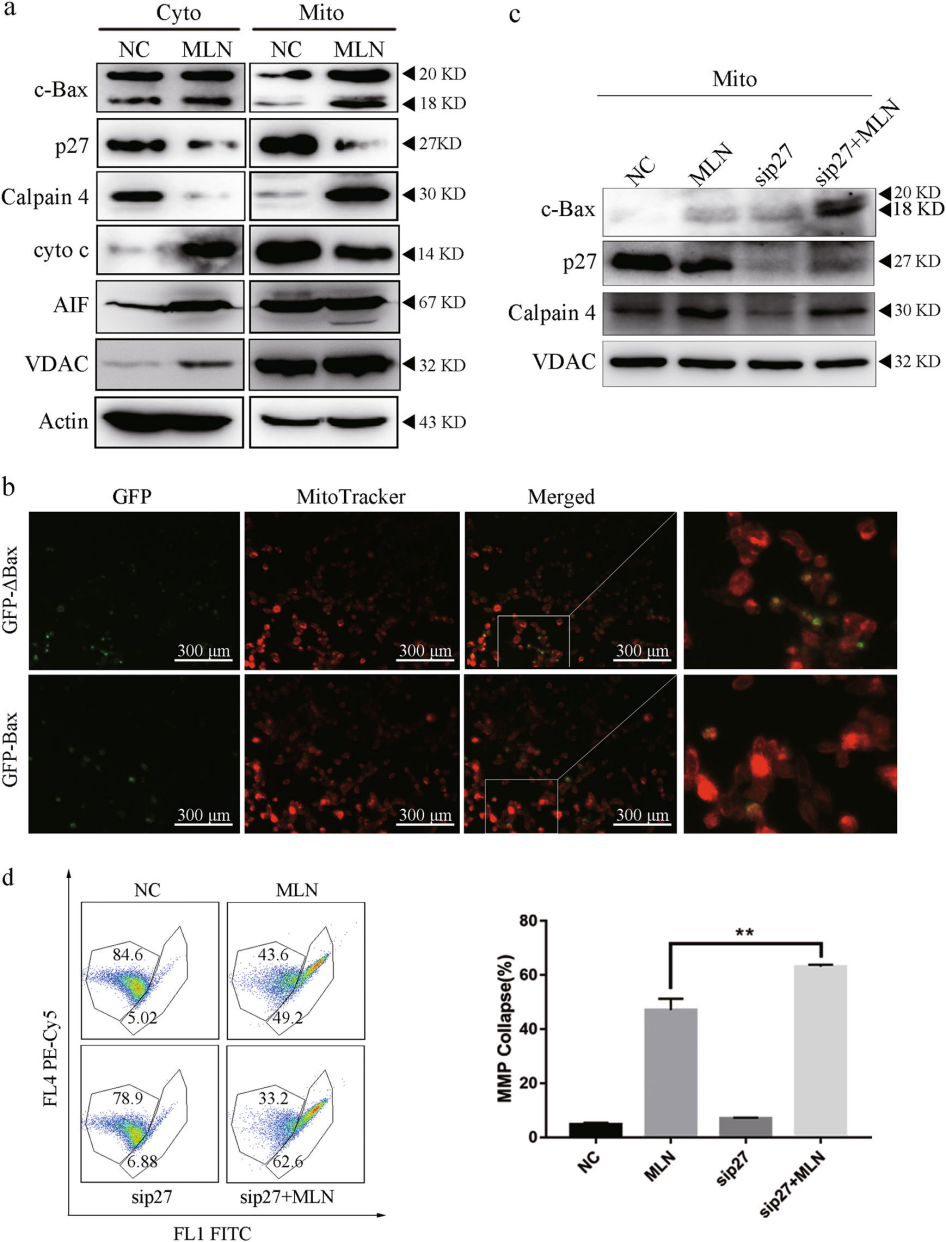
c-Bax localized to mitochondria and induced cytochrome c release and led to apoptosis. **a** Mitochondrial isolation to detect c-Bax distribution and cytochrome c release upon 200 nM MLN8237 treatment for 72 h in AGS. Mitochondrial isolation was conducted according to the manufacturer protocol and measured indicated protein expression in the various component of the cell lysate. **b** Location detection of EGFP-Bax and EGFP-ΔBax in 293 T. EGFP-Bax or EGFP-ΔBax was transfected into 293 T and photographed through a fluorescence microscope before incubated with mitotracker for 15 min at 37 °C. **c** Enhanced c-Bax localized to the mitochondrial after p27 silencing in 200 nM MLN8237-treated AGS cell line for 72 h. Cells were subjected to mitochondrial isolation and immunoblotting for quantification of mitochondrial c-Bax. **d** Augmented deterioration of the mitochondrial membrane potential (MMP) by p27 silencing in MLN8237-treated AGS cell line. AGS cells transfected with negative control or p27 siRNA were treated with 200 nM MLN8237 for 72 h, and subjected to JC-1 staining and FCM to detect MMP collapse. Error bars represent SD from three independent experiments. Representative microscopic images are shown. This immunoblot is representative of three independent experiments. Asterisk (*) indicates a significant difference. **P < 0.01 two-tailed Student’s *t* test

Bax is a member of the Bcl-2 family and core regulator of the intrinsic pathway of apoptosis. Upon stimulation, Bax are activated and oligomerize at the outer mitochondrial membrane to mediate its permeabilization and allows the release of pro-apoptosis factors such as cytochrome c and SMAC/DIABLO from the mitochondria to cytosol to activate the caspase cascade, which is considered as a key step in apoptosis^20^. To explore the effect of c-Bax on apoptosis, we separated the mitochondrial component of the cell lysates in AGS. We found that c-Bax could localize to mitochondria and promote its cleavage on appearance of calpain 4 protein. The localization of c-Bax to the mitochondria induced the release of cytochrome c and AIF (Fig. 4a). The intrinsic apoptosis pathway is initiated with Bax localization to mitochondria. We thus decided to investigate the localization of c-Bax in 293 T cells transfected with the plasmid encoding GFP-tagged Bax (GFP-Bax) or c-Bax (GFP-ΔBax). Compared with GFP-Bax, GFP-ΔBax exhibited strong mitochondrial staining patterns (Fig. 4b). Compared with MLN8237 treatment, p27 silencing increased Bax cleavage by calpain, however no calpain accumulation were observed in mitochondrial component in combined treatment (Fig. 4c). Taken together, like Bax, c-Bax localizes to mitochondria and induces apoptosis. After silencing p27, the MLN8237-treated cells underwent a higher MMP collapse and increased apoptosis (Fig. 4d). These results indicated that MLN8237-induced c-Bax localized to mitochondria and led to mitochondrial apoptosis. p27 knockdown had a synergistic effect in MLN8237-induced MMP collapse in gastric cancer cells.

### Bcl-2 cannot block apoptosis induced by c-Bax

**Fig. 5:**
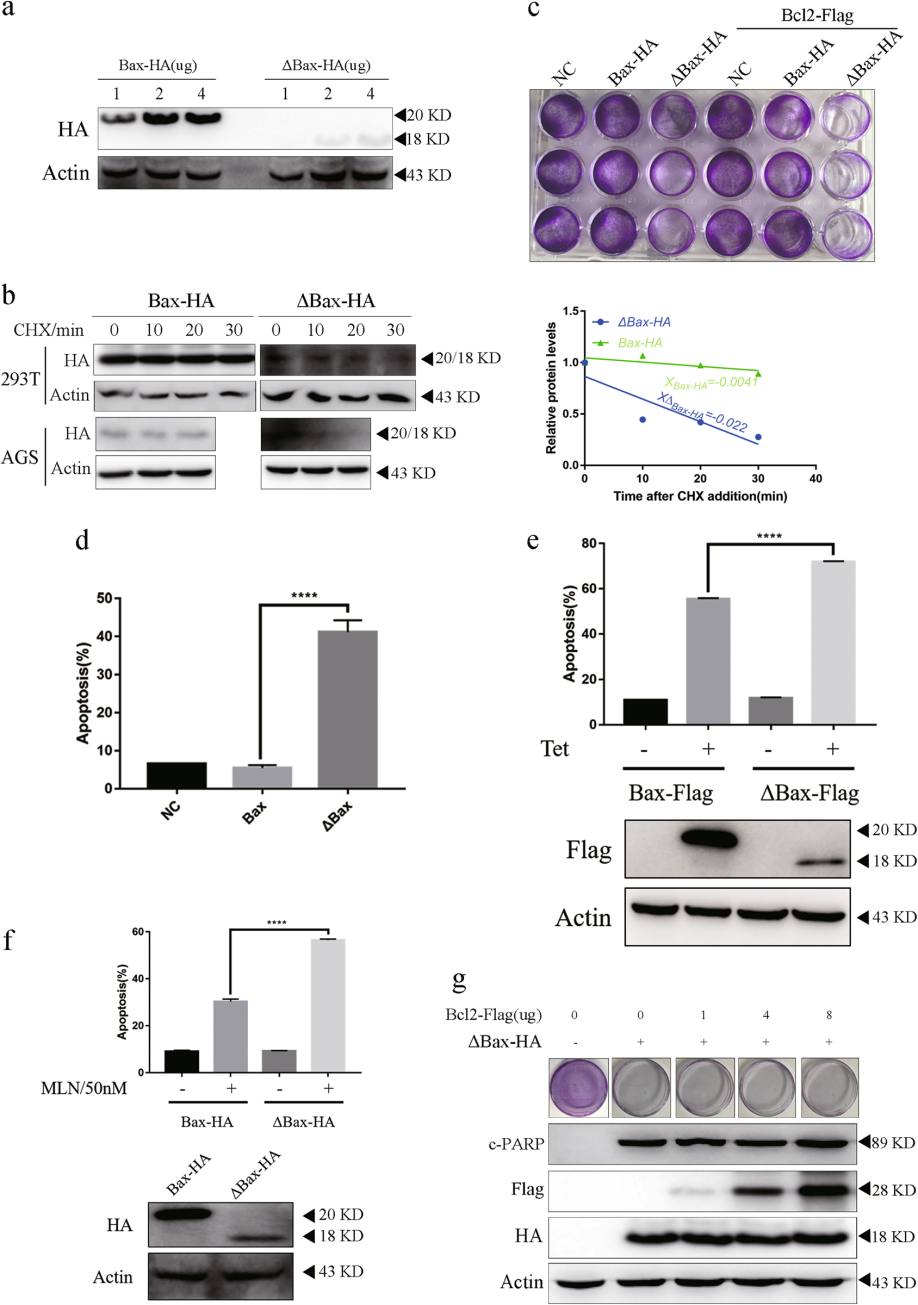
Bcl-2 cannot block apoptosis induced by c-Bax. **a** Differential expression of Bax-HA and ΔBax-HA. AGS cells were transfected with 1 µg, 2 µg, or 4 µg of either Bax-HA or ΔBax-HA for 24 h and subjected to immunoblotting for protein expression. **b** Short-lived ΔBax-HA than Bax-HA. CHX (50 ug/ml) was added to 293 T or AGS cells transfected with plasmid encoding Bax-HA or ΔBax-HA prior to treatment and cultured for 24 h. Equivalent lysates from the cells harvested at the indicated time points were immunoblotted for indicated protein expression. **c**, **d**, **g** Bcl-2 did not decrease apoptosis induced by ΔBax-HA. 1 µg Bax-HA or 4 µg ΔBax-HA plasmid was transfected with 4 µg Bcl-2-Flag (**c**) or 4 µg ΔBax-HA and indicated quality of Bcl-2-Flag plasmid, then cultured for 24 h and AGS subjected to crystal violet staining or immunoblot (**c**, **g**). AGS cells were treated with 1 µg Bax-HA or 4 µg ΔBax-HA plasmid (**d**) and subjected to Annexin V/propidium iodide double staining and FCM to detect apoptosis. **e**, **f** Cleaved Bax was a stronger apoptosis inducer. **e** The tet-on system was constructed and protein expression was induced by 10uM Tetracyclines for 24 h in AGS. Apoptosis level and protein level was detected by FCM and immunoblot. **f** The AGS stable cell line was constructed and incubated with 50 nM MLN8237 for 72 h. Apoptosis level and protein level was detected by FCM and immunoblot. Error bars represent SD from three independent experiments. This immunoblot is representative of three independent experiments. Asterisk (*) indicates a significant difference. ****P < 0.0001 two-tailed Student’s *t* test

We did not know what the potential role of c-Bax, a truncated fragment of Bax, would be applied in cancer therapeutics. Therefore, we cloned Bax and N33- cleaved Bax (ΔBax) into pcDNA3.1 plasmids and transfected into cells. To our surprise, Bax and ΔBax expression levels were unequal when equal mass of each plasmid were used in transfection (Fig. 5a). As they have the same plasmid backbone, we expected ΔBax to have a shorter half-life. Unlike Bax-HA, ΔBax-HA was rapidly eliminated by protein synthesis inhibitor CHX in 293 T and AGS cell lines (Fig. 5b), providing a strong evidence that c-Bax is a short-lived protein that can be constitutively degraded by proteasomes. The half-life of ΔBax-HA in 293 T was estimated to be ~17 min under these experimental conditions (Fig. 5b).

To investigate how the functions of ΔBax were different from that of Bax, we transfected 1 μg of Bax-HA plasmid and 4 μg of ΔBax-HA plasmid into gastric cancer cells. We found that ΔBax was a stronger apoptosis inducer than Bax at a low protein expression level. Bax had no significant function in inducing apoptosis in gastric cancer cells (Fig. 5c, d). However, higher transfected Bax-HA plasmid also induced apoptosis in 293 T and AGS cell lines (Figure S10a, b). To reinforce the conclusion that cleaved Bax was a stronger apoptosis inducer, we made an inducible AGS cell line where the expression of Bax and its cleaved form can be turned on. As shown in Fig. 5e, the cleaved form of Bax induced more apoptosis than total wide type (WT). We also constructed Bax-HA and ΔBax-HA stable AGS cell line. 50 nM MLN8237 treatment induced increased percentage of apoptosis in ΔBax-HA stable cell line than Bax-HA stable cell line. Above all, we could illustrate that cleaved form of Bax is a stronger apoptosis inducer than Bax.

Bax, as the key molecule for inducing the mitochondrial apoptosis pathway, can be blocked by Bcl-2. However, Bcl-2 did not block c-Bax-dependent apoptosis when Bcl-2 was overexpressed (Fig. 5c). To further solidify our conclusions, we transfected 4 μg ΔBax-HA plasmid with increasing Bcl-2 plasmid. Bcl-2 still did not block apoptosis, even increasing to 8 μg of Bcl-2 plasmid transfection into the cells (Fig. 5g). Put together, we can state that c-Bax is a stronger apoptosis inducer and c-Bax-dependent apoptosis cannot be blocked by Bcl-2-the canonical Bax inhibitor.

### Both AURKA and p27 were simultaneously overexpressed in gastric cancer tissues and its increased expression proved to be a poorer prognosis

**Fig. 6:**
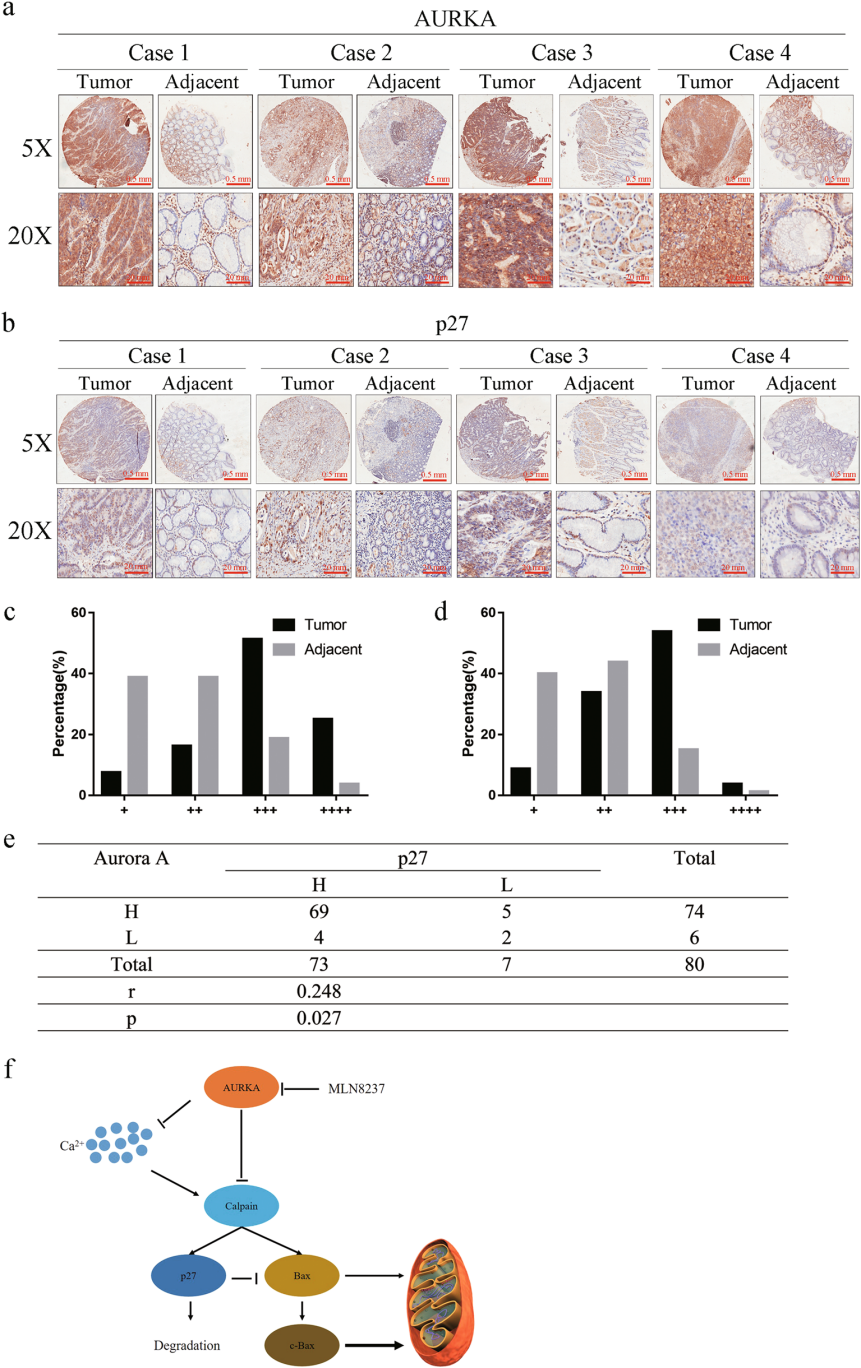
AURKA and p27 were overexpressed in tumor tissues and had a positive correlation in late TNM stage of gastric cancer tissues. **a**–**d** IHC staining of human gastric tumor tissues and adjacent tissues using specific AURKA and p27 antibodies, respectively. Samples were classified according to the intensity of the staining of AURKA and p27 expression. **e** A four-fold square showed the correlations of AURKA and p27 expression in gastric cancer. **f** Working model of the suppression of AURKA in gastric cancer cells relieved inhibition of Bax cleavage by p27 and induced more intensive apoptosis

To further investigate the relationship between AURKA and p27, we analyzed AURKA and p27 expression by immunohistochemistry (IHC) in gastric cancer tissue samples with matched adjacent tissues from 80 patients. Both AURKA and p27 were highly expressed in the tumor tissues compared with the adjacent tissues (Fig. 6a–d). Based on the IHC staining intensity, the samples were classified into four groups from weakest staining designed group 1 (+) to strongest staining designated group 4 (++++). The staining intensity of group 1 was defined as low expression of the protein and the staining intensity of other groups was defined as high expression. In group 1, the majority of adjacent tissues had weak expression of AURKA (31/80) compared with that of tumor tissues (6/80); however, in the other groups, the majority of gastric cancer tissues (74/80) had stronger expression of AURKA compared with that of adjacent tissues (49/80). Similar results were also observed for p27 expression in the same cohort. Importantly, high expression of AURKA and p27 exhibited a positive linear correlation in the gastric cancer tissue samples (*r* = 0.248, *P* = 0.027, Fig. 6e).

We classified the patients into three groups based on the AURKA and p27 expression: both AURKA and p27 high expression group (AURKA^H^p27^H^), either AURKA or p27 high expression group (AURKA^H^p27^L^/AURKA^L^p27^H^) and both AURKA and p27 low expression group (AURKA^L^p27^L^). Of the patients in the study, 86.2 % (69/80) were in the AURKA^H^p27^H^ group. Patients with high AURKA and p27 expression had a late TNM stage (*p* = 0.031), which indicated a worse prognosis (Table 1). However, gender, age, tumor volume, tumor sites, clinical pathological features and invasion stage had no significant correlations to AURKA and p27 expression (Table 1). Our results suggest that the higher expression of AURKA and p27 in gastric cancer play an important role in gastric cancer carcinogenesis.

**Table 1:** Clinical characteristics of 80 gastric cancer patients and AURKA/p27 expression

		Aurora A and p27 expression			*P*
		2H	1H1L	2L	
Sex					
Male	63	54	7	2	0.191
Female	17	15	2	0	
Age (years)					
<60	27	22	5	0	0.071
>=60	53	47	6	0	
Tumor volume (cm^3^)					
<=5	46	40	6	0	0.203
>5	34	29	3	2	
Tumor location					
Up	19	15	4	0	0.687
Middle	22	19	3	0	
Down	39	35	4	0	
Pathological grade					
II	26	20	6	0	0.991
II–III	25	25	0	0	
III	29	24	3	2	
Invasion stage					
Mucosal and submucosal	1	1	0	0	0.372
Muscular layer	8	7	1	0	
Serous membrane and outmembrane	71	61	8	2	
Lymph node metastasis					
0	17	16	1	0	0.510
1–6	36	29	7	0	
>=7	27	24	3	0	
TNM stage					
I	5	5	0	0	0.031
II	22	19	3	0	
III	46	41	3	2	
IV	7	4	3	0	
Total	80				80

*P* < 0.05 was considered statistically significant. Pearson’s chi-square test was used

## Discussion

There are 14 known human calpain isoform genes^18^, among which μ-calpain and m-calpain, are the two most studied, and which were named on the basis of the concentration of calcium ions required for their activity in vitro^21^. Both μ-calpain and m-calpain are heterodimers consisting of a different catalytic subunit and the same regulatory subunit (CAPNS1). Calpain-mediated cleavage can either result in protein turnover or generate functional truncated proteins. Calpain expression is altered during tumorigenesis, and the proteolysis of numerous substrates, such as IκB, focal adhesion proteins and proto-oncogenes (for example, Myc), has been implicated in tumor pathogenesis. Increased expression of calpain is known to influence the cellular response to cancer therapies, providing justification for the development of calpain activators or inhibitors. There are relationships between calpain deficiencies and a variety of defects, including lethality^22^, muscular dystrophies^23^, gastropathy^24^, diabetes^25^, and tumorigenesis^26^. In this study, we showed that degradation of p27 by calpain followed by cleavage of Bax induces acute apoptosis. Cleaved Bax is able to induce apoptosis much more intensively than full-length Bax. Bcl-2, a Bax inhibitory protein, has no effect on the apoptosis induced by cleaved Bax.

p27, a classical tumor suppressor protein, is thought to be transported to nucleus where it binds to the CDK–Cyclin complex to inhibit the cell cycle. Unlike other cell cycle inhibitors such as p16 and p21, which are frequently mutated or deleted in human cancers, genetic alterations of p27 are rare. Rather, p27 is dysregulated in cancers by transcriptional and post-translational mechanisms. Low levels of p27 correlate with poor prognosis and survival in many types of cancer^10,27^. Surprisingly, p27+/− mammary epithelium is more susceptible to oncogene-induced tumorigenesis compared to p27-null glands^28^. Similarly, triple mutants that are heterozygous for a p27 null allele(Nkx3.1+/− or −/−; Pten+/−; p27+/−) display enhanced prostate carcinogenesis, whereas mice that are homozygous null for p27(Nkx3.1+/− or −/−; Pten+/−; p27−/−) show inhibition of cancer progression^29^. Mislocalization of p27 to the cytoplasm of breast cancer cells confers resistance to anti-HER2 target therapy^30^. Thus, we believe that p27 was localized to the cytoplasm in AGS gastric cancer cells, which facilitated the p27 degradation thereby diminishing its protective role and leading to apoptosis. When treated with MLN8237, p27 was degraded by calpain, rather than by Skp2, a canonical p27 E3 ligase. Thus, the cytoplasmic p27 level, or the cytoplasmic to the nuclear ratio of p27 levels may be a better predictor of prognosis than nuclear p27 in tumor patient.

The Bax protein consists of 9 α-helixes. Bax is activated during apoptosis as a consequence of changes of its conformation, which is believed to be mainly regulated by the intramolecular interaction between its N- and C-terminal regions^31–33^. Structural biology experiments suggests that the α-9 helix normally resides in a canonical hydrophobic groove of Bax(formed by helices α3–5) when the protein is inactive^34^. The N-terminal α1 helix of Bax keeps the α9 helix engaged in the dimerization pocket, rendering Bax inactive in the cytosol. Apoptosis activators, such as tBid, Bim, and Puma, attack and expose the α1 helix of Bax, resulting in secondary disengagement of α9 helix and thereby mitochondrial insertion, and release of mitochondrial component into the cytoplasm^35^. N-terminal-truncated Bax at the 33th glutamine may have auto-activated ability to provoke apoptosis. depending on their BH3 domain, Bax can combine with Bcl-2 to inhibit cell apoptosis. To our surprise, Bcl-2 could not suppress apoptosis induced by c-Bax, which has BH3 domain. Till now, we have no idea about the concrete functions of c-Bax. Mitochondrial pores formed by Bax are favorable for the exit of mitochondrial components, such as cytochrome c, AIF. It may be the mitochondrial pore formed by c-Bax is bigger in diameter than that formed by Bax, which would lead to a quicker export of mitochondrial components. Drugs, which can induce c-Bax generation, such as inhibition of AURKA by MLN8237, probably have a better response in Bcl-2 driven cancers.

Inhibition of AURKA led to strong cytotoxicity to gastric cancer cells with cytoplasmic p27 degradation and Bax cleavage, which were caused by activating the calpain pathway. Further, p27 knockdown synergistically increased the inhibition of proliferation and increased c-Bax-induced apoptosis. Importantly, c-Bax is a high-efficiency apoptosis-inducing factor and could not be suppressed by the canonical inhibitory factor, Bcl-2. Targeting AURKA expression or activity by inducing p27 degradation and Bax cleavage provided a promising new anti-cancer therapeutic. This illustrates that the chemotherapeutic agents which could induce c-Bax expression could be applied to Bcl-2-driven tumor cells and patients.

## Materials and methods

### Cell culture and reagents

The human gastric cancer cell line, AGS, was achieved from the Type Culture Collection of the Chinese Academy of Science (Shanghai, China). AGS cells were cultured in F-12K medium (Gibco, Life Technologies, Carlsbad, CA, USA), containing 10% fetal bovine serum (Gibco, Life Technologies, Carlsbad, CA, USA) and 1% penicillin-streptomycin with 5% CO_2_ at 37 °C. MLN8237 (Alisertib) was purchased from Selleck (Shanghai, China). Annexin V, FITC Apoptosis Detection Kit was purchased from Dojindo (Shanghai, China).

### Immunoblotting

Cell lysates were extracted with cell lysis buffer (Beyotime Biotechnology, Nantong, China), and protein concentration was measured by BCA Protein Assay Kit (Tiangen CO., LTD, China). At least 20 μg of each protein sample was loaded for immunoblotting and tested by antibodies that against p27, p21, Skp2, c-PARP, c-caspase 3, c-caspase 9, cytochrome c, AURKA, AURKB (Cell Signaling Inc., Danvers, MA, USA), c-Bax, calpain 4 (Santa Cruz Biotechnology, Santa Cruz, USA), AIF (Epitomics, Hanghzou, China), HA-tag, Flag-tag, Actin (Cw Biotech, Shanghai, China).

### Immunoprecipitation(IP)

For IP, cells were harvested after washed twice by cold PBS and then lysed with 0.5% NP-40 for 10 min on ice. The lysates were centrifuged at 12,000 rpm for 15 min and then incubated with anti-HA mAb or anti-Flag conjugated beads(Sigma, USA) for 2–4 h at 4 °C. IP beads were washed four times with lysis buffer and resuspended with 1 × loading buffer and analyzed by SDS-PAGE followed by immunoblot.

### FACS analysis

Cells were harvested at specific time points and fixed overnight at −20 in 70% ethanol. Then stained with propidium iodide (36 μg/ml, Sigma) containing 400 μg/ml RNase (Roche, Mannhein, Germany) for 30 min and cell cycle analysis was conducted by flow cytometry (CyAn ADP, Beckman Coulter, Bera, CA, USA). To measure apoptosis, mitochondrial membrane potential and Calcium influxes, cells were harvested and stained with FITC Apoptosis Detection Dye, JC-1, Fluo-3 AM (Beyotime Biotechnology), and then analyzed by flow cytometry. The data analysis of cell cycle was processed by ModFit software and other FACS data were processed using FlowJo software.

### RNA Interference and plasmid transfection

siRNA oligonucleotides were synthesized by RiboBio (Guangzhou, China). Bax siRNA was synthesized by Santa Cruz Biotechnology (Santa Cruz, USA). The human p27 and Bax cDNA were cloned into pcDNA 3.1 vector with either a Flag or HA tag. Both of the siRNA and plasmids were transfected into gastric cancer cells using Lipofectamine RNAiMAX or Lipofectamine 2000 (Invitrogen, USA), respectively, according to the manufacturer’s instructions. The stable Bax-HA or ΔBax-HA cell line was screened with 10 μM puromycin treatment.

For the construction of Tet on system, Bax or ΔBax was cloned into pTRIPZ plasmid. pTRIPZ-Bax/ΔBax was transfected into AGS cell line and the stable cell line was screened with 10 μM puromycin treatment. The overexpression of Bax or ΔBax was induced by 10 μM Tetracyclines.

### MTS and Hoechst 33342 assays

Gastric cancer cells were seeded into 96-well plates at 2000 cells per well in triplicate for MTS assay (Promega, USA), according to the manufacturer’s specification and measured by TECAN Infinite 200 Pro plate reader (Switzerland). For the Hoechst 33342 assay, gastric cancer cells were seeded into 60-mm dishes, and washed twice with PBS. The cells were incubated in Hoechst 33342 for 15 min at 37 °C and then photographed under a fluorescence microscope (Leica, Germany).

### Isolation of mitochondria

The mitochondrial and cytoplasmic cell lysate fructions were isolated according to the manufacturer’s protocol (Beyotime Biotechnology, China). Both fractions of lysate were analyzed by SDS–PAGE and immunoblotting.

### Statistical analysis

Graph Pad Prsism 5.0 was used to calculate the significant difference between groups. The unpaired two-tail *t*-test was used for comparisons between two groups. *P* values < 0.05 were considered significant.

## Electronic supplementary material

Figure S1

Figure S2

Figure S3

Figure S4

Figure S5

Figure S6

Figure S7

Figure S8

Figure S9

Figure S10

Supplementary figure legends
