# Supplementary figures and images for: Suppression of AURKA alleviates p27 inhibition on Bax cleavage and induces more intensive apoptosis in gastric cancer
Source: Cell Death Dis. 2018 Jul 16;9(8):781. doi: 10.1038/s41419-018-0823-3 (PMC6048174; doi:10.1038/s41419-018-0823-3)

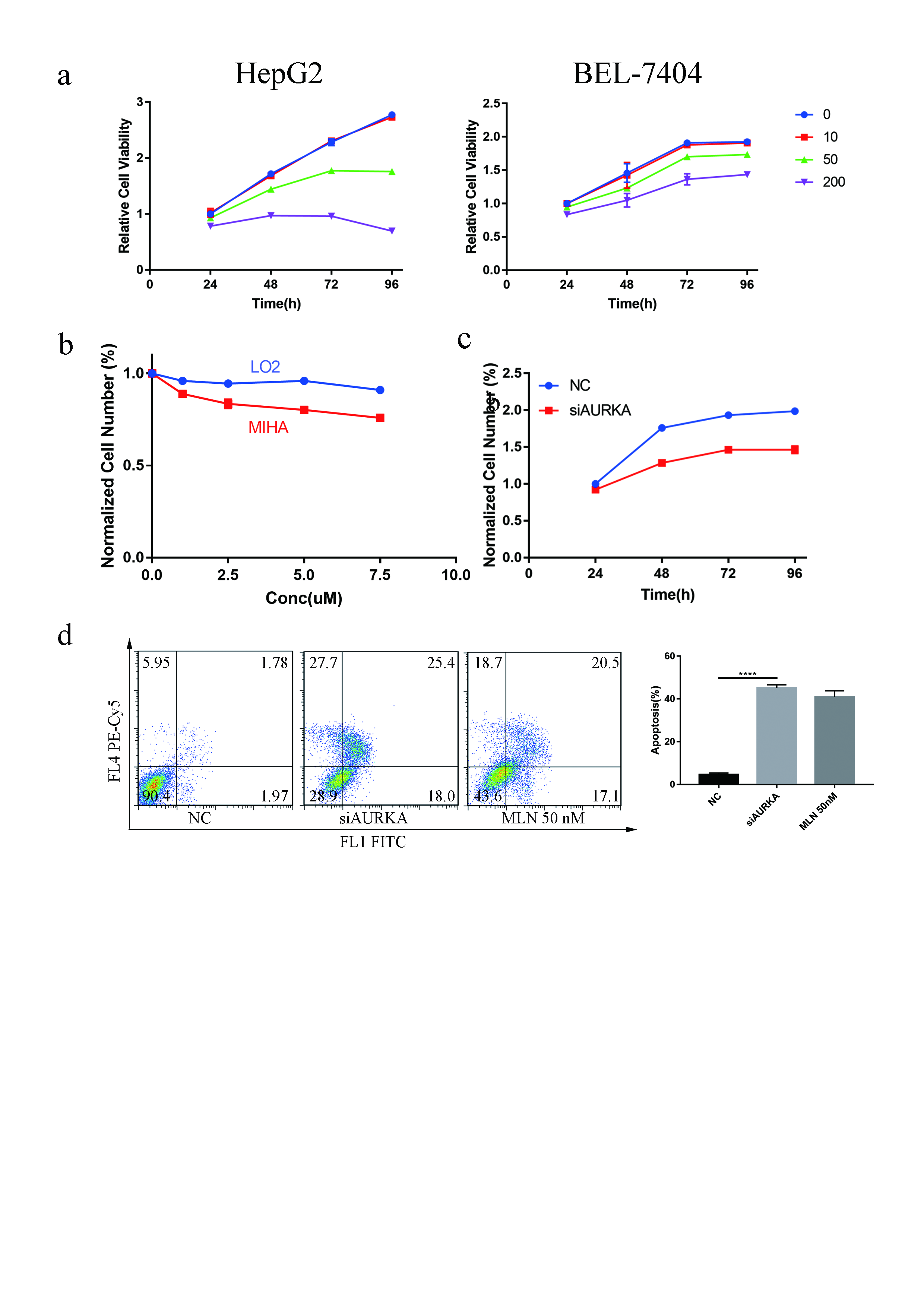

Supplement: Supplementary file 1 — Figure S1 [file 41419_2018_823_MOESM1_ESM.tif]

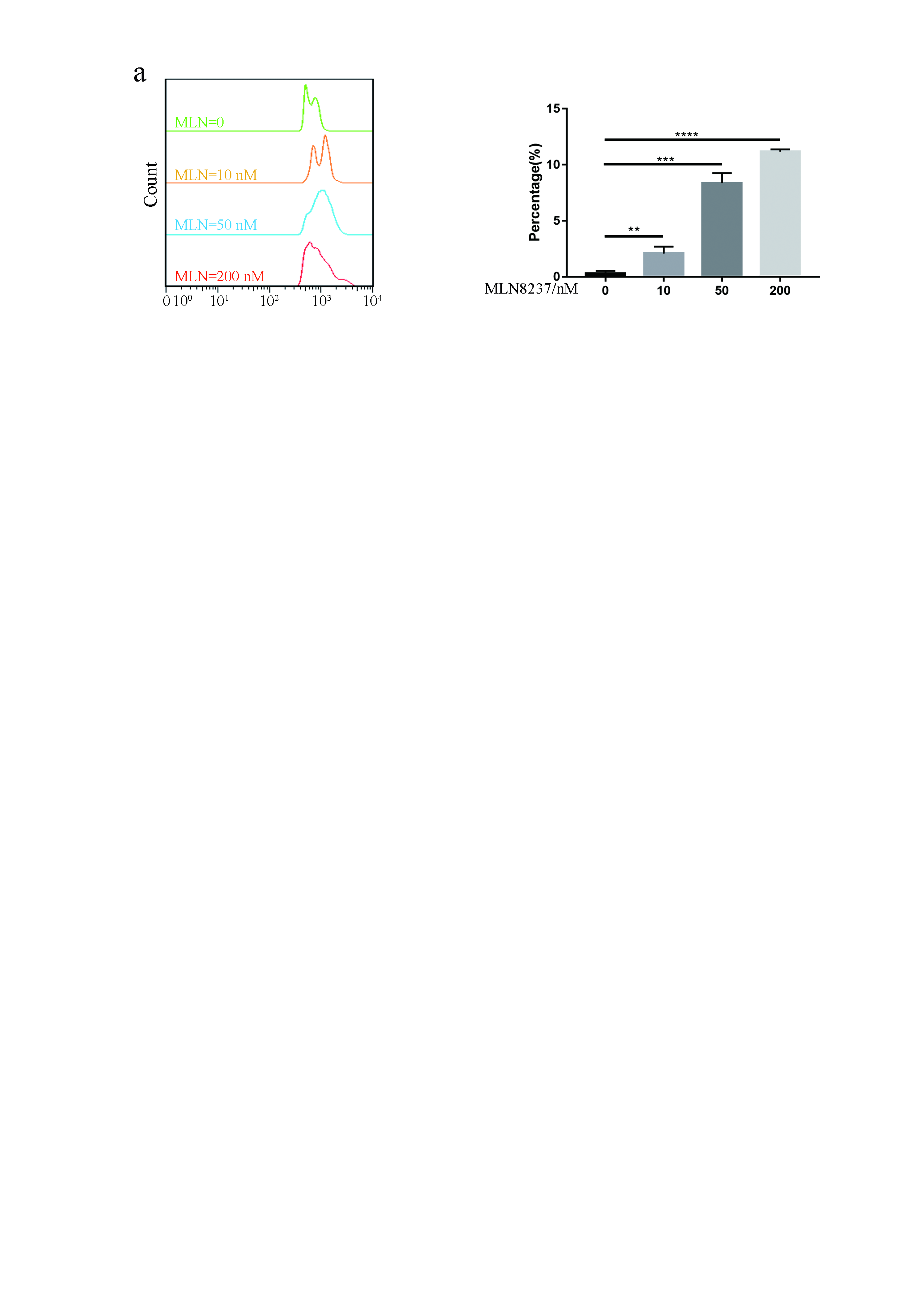

Supplement: Supplementary file 2 — Figure S2 [file 41419_2018_823_MOESM2_ESM.tif]

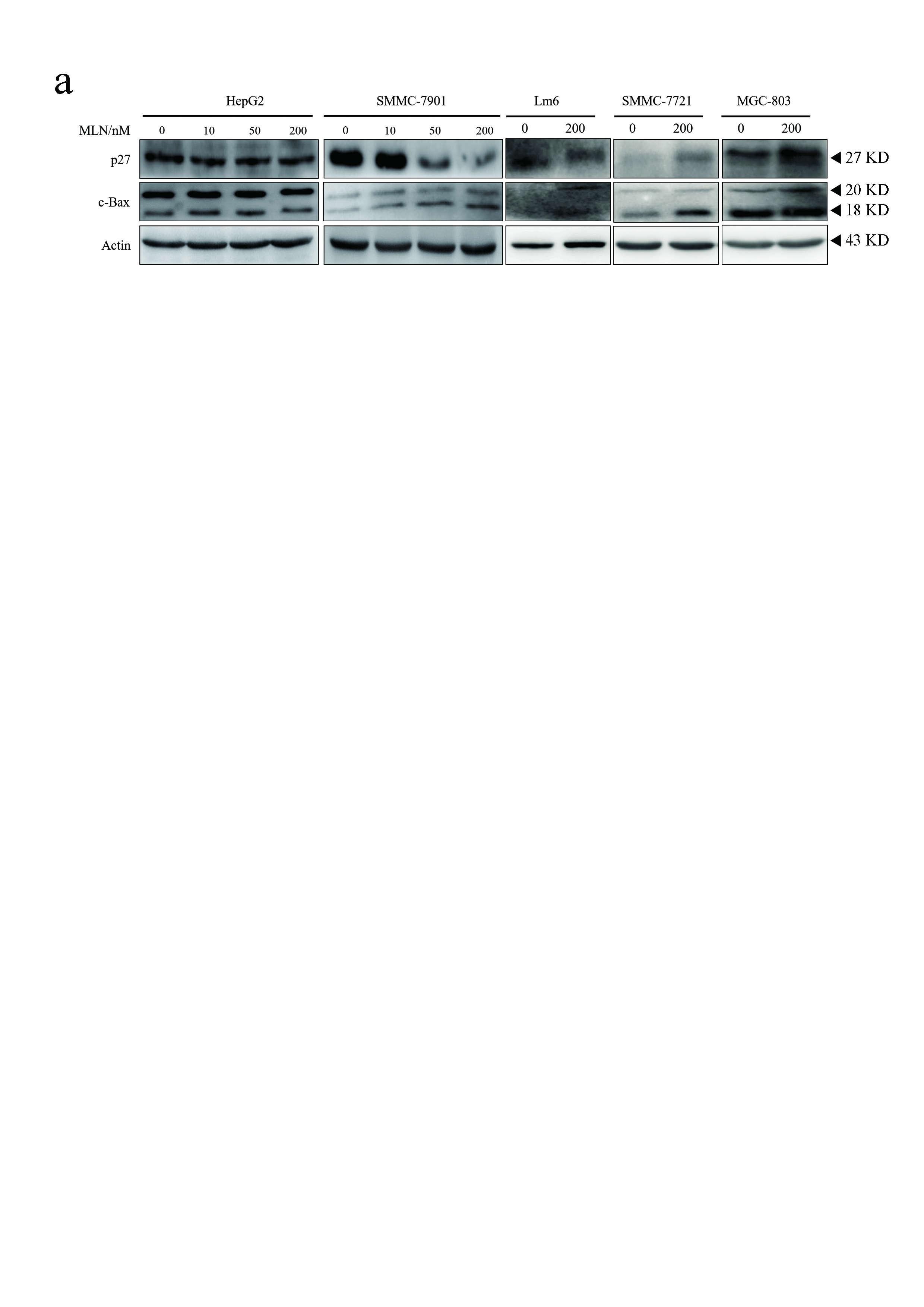

Supplement: Supplementary file 3 — Figure S3 [file 41419_2018_823_MOESM3_ESM.tif]

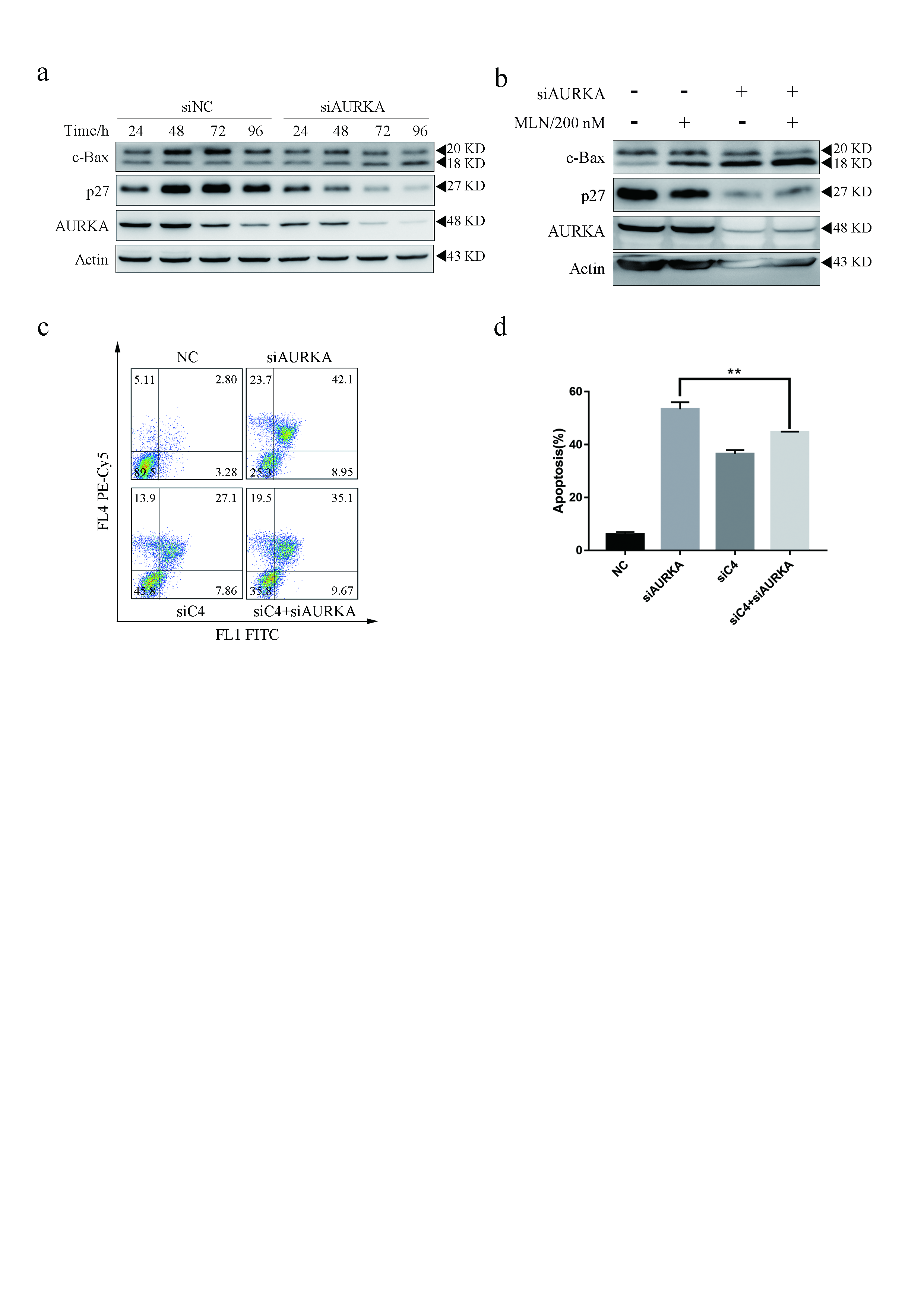

Supplement: Supplementary file 4 — Figure S4 [file 41419_2018_823_MOESM4_ESM.tif]

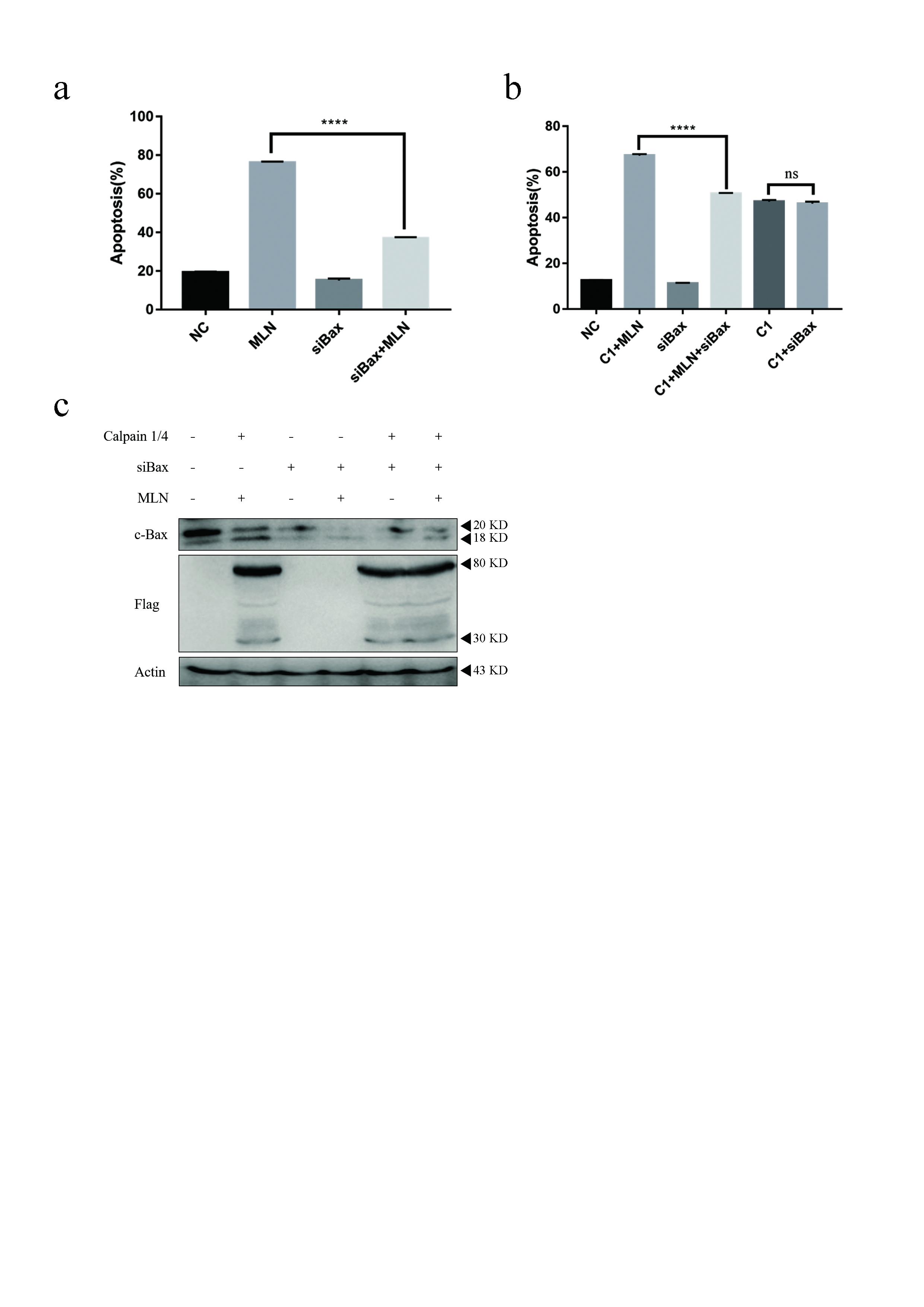

Supplement: Supplementary file 5 — Figure S5 [file 41419_2018_823_MOESM5_ESM.tif]

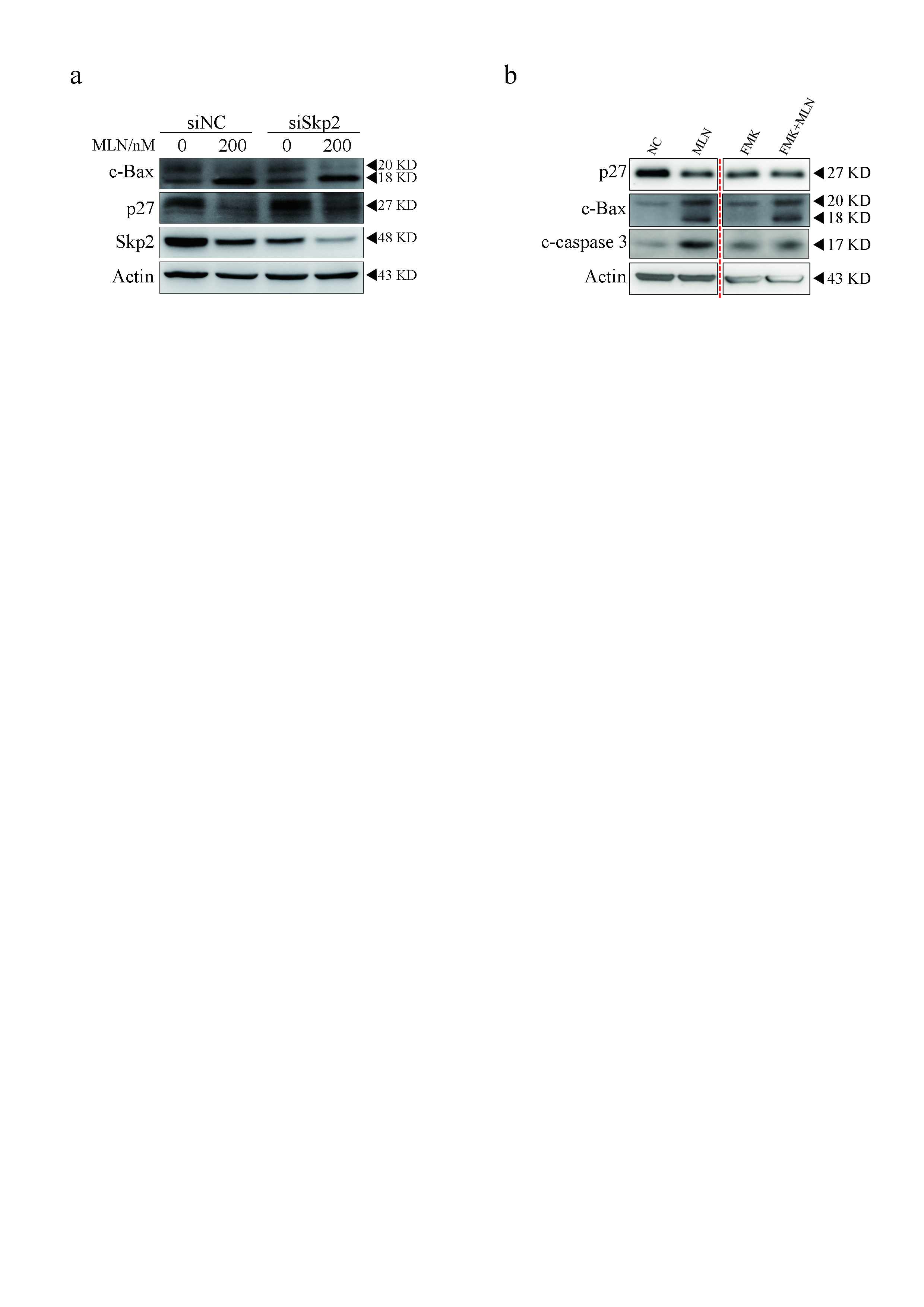

Supplement: Supplementary file 6 — Figure S6 [file 41419_2018_823_MOESM6_ESM.tif]

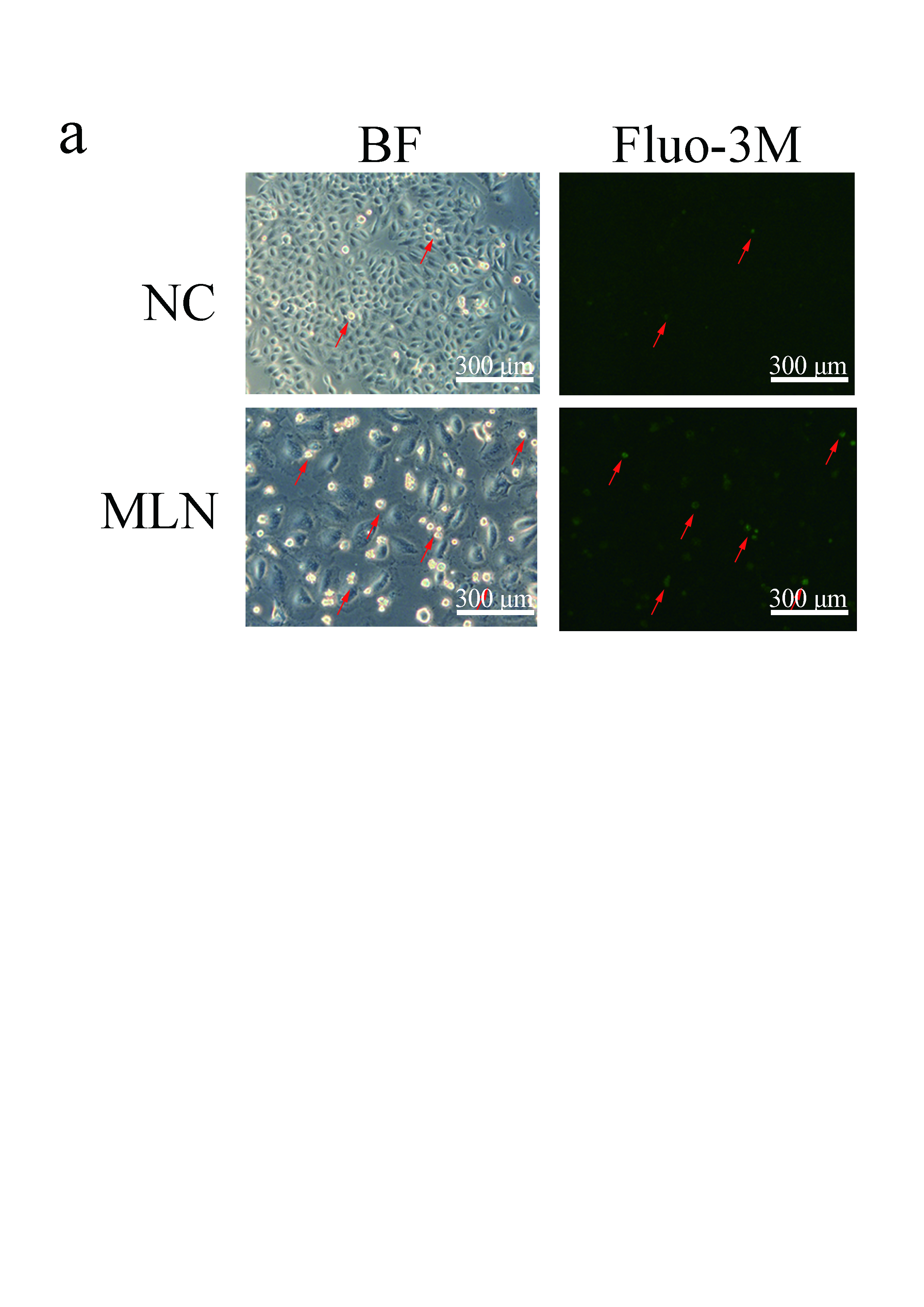

Supplement: Supplementary file 7 — Figure S7 [file 41419_2018_823_MOESM7_ESM.tif]

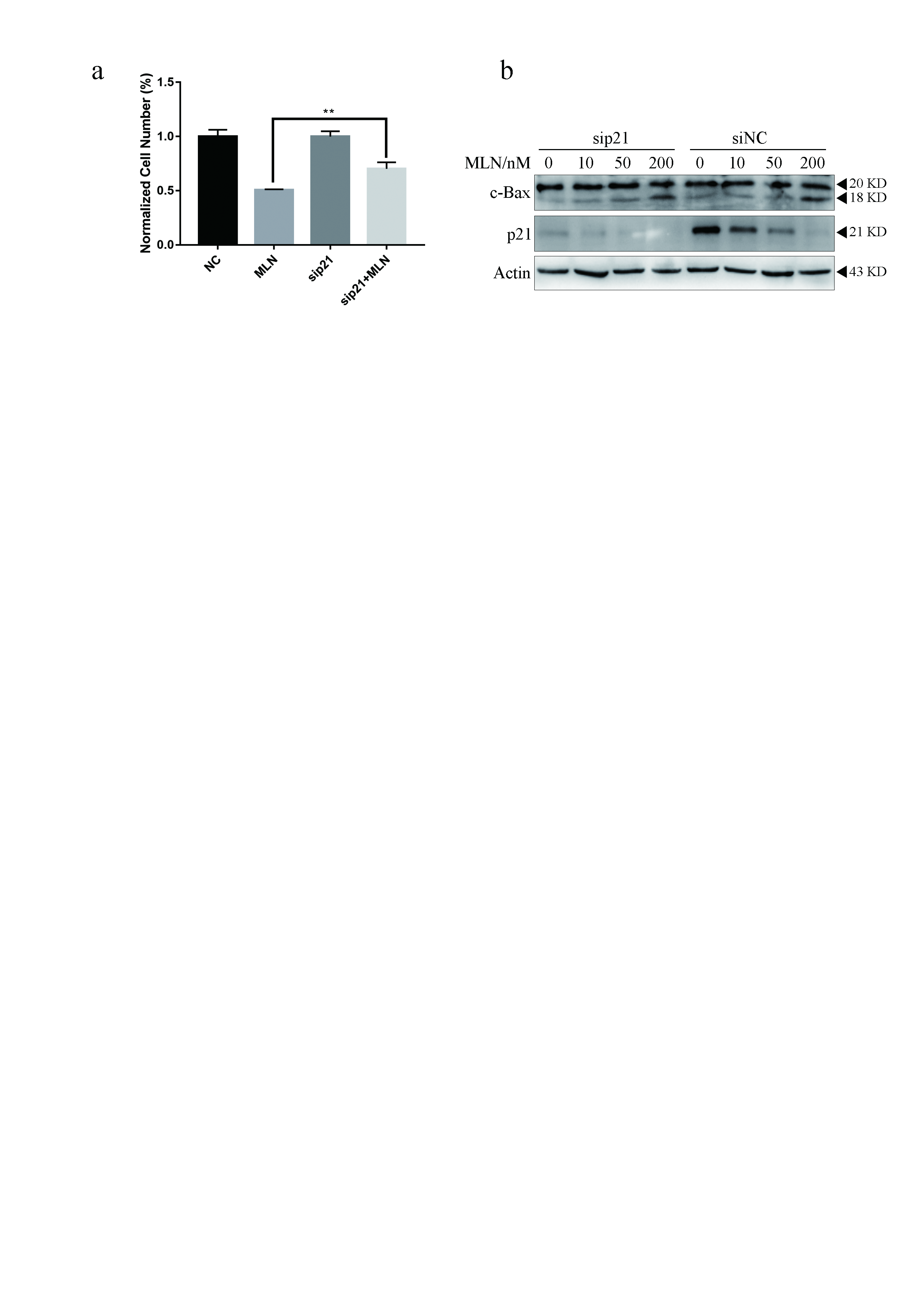

Supplement: Supplementary file 8 — Figure S8 [file 41419_2018_823_MOESM8_ESM.tif]

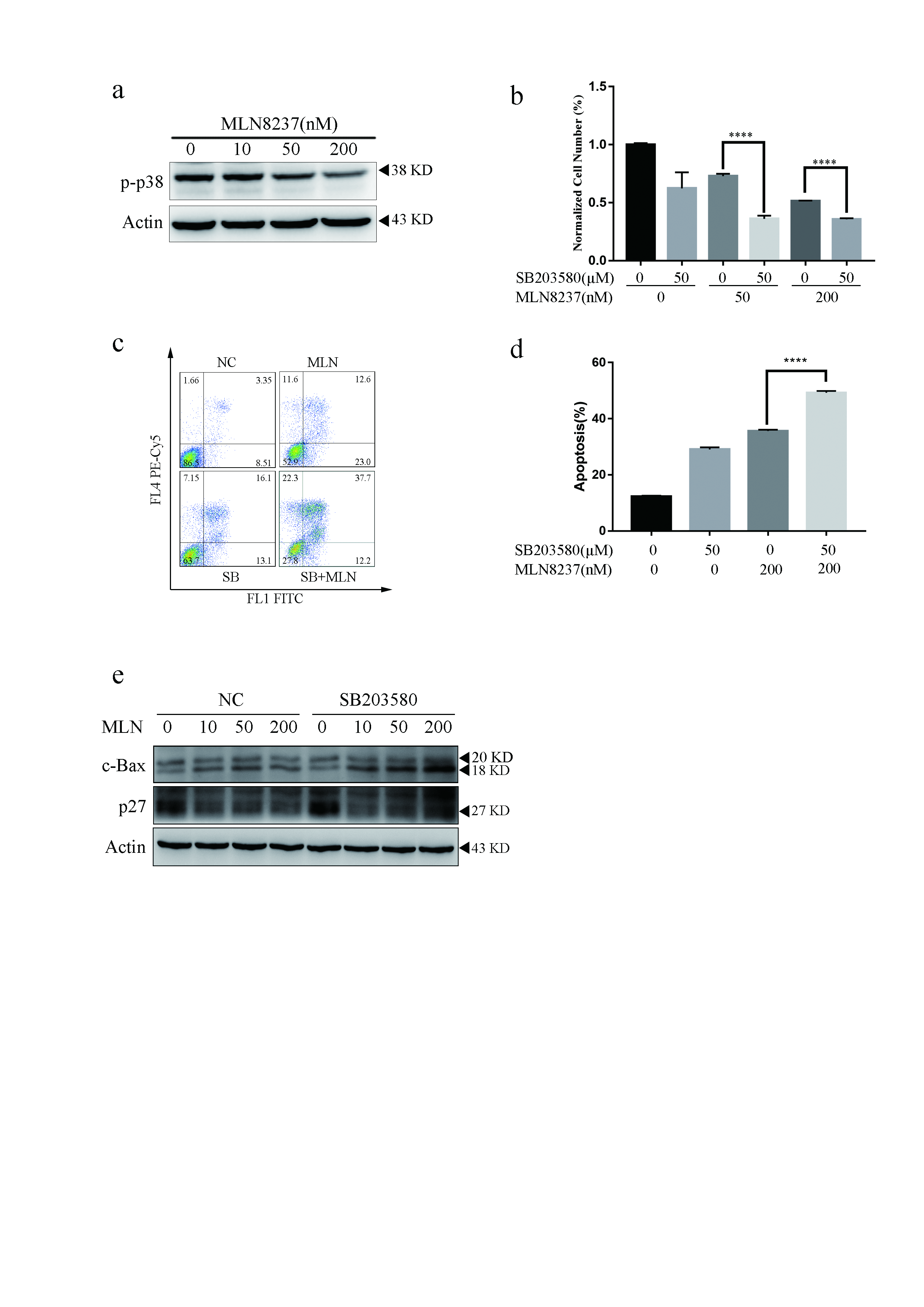

Supplement: Supplementary file 9 — Figure S9 [file 41419_2018_823_MOESM9_ESM.tif]

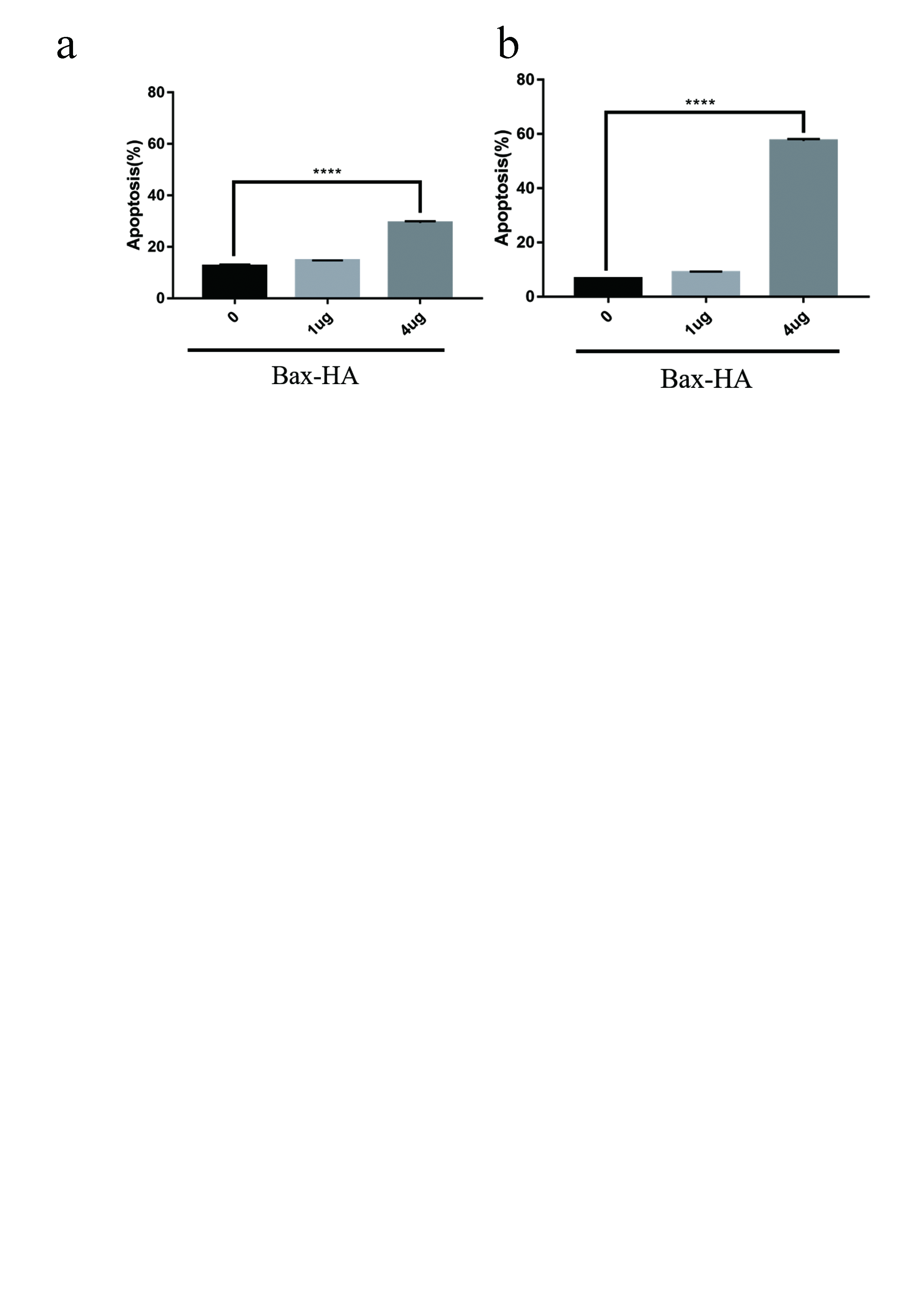

Supplement: Supplementary file 10 — Figure S10 [file 41419_2018_823_MOESM10_ESM.tif]
