# Supplementary material for: Suppression of AURKA alleviates p27 inhibition on Bax cleavage and induces more intensive apoptosis in gastric cancer
Source: Cell Death Dis. 2018 Jul 16;9(8):781. doi: 10.1038/s41419-018-0823-3 (PMC6048174; doi:10.1038/s41419-018-0823-3)
Supplement: Supplementary file 11 — Supplementary figure legends [file 41419_2018_823_MOESM11_ESM.docx]

Figure S1

Cell cytotoxicity of hepatoma cells treated with MLN8237. (a) MLN8237 suppressed the proliferation of hepatoma cells. HepG2 and BEL-7404 were treated with MLN8237 at the indicated concentration for 24-96h, then cell number was counted with MTS assay. (b) Hepatocyte cell lines tolerated MLN8237-induced cytotoxicity. LO2 and MIHA cells were treated with MLN8237 at the indicated concentration for 24-96h, and cell number were counted with MTS assay. The mean and SDs of the plots were obtained from 3 wells within 3 independent MTS assays. (c-d) Suppression of AURKA repressed the activity of gastric cancer cells. Cells were transfected with negative control or AURKA siRNA for 24-96hrs, and (c) cell proliferation was analyzed by MTS assay and (d) Apoptosis detection by Annexin V/propidium iodide double staining. (e) MLN8237 treatment led to p27 downregulation and Bax cleavage in other cancer cell types. All cells were treated with indicated MLN8237 concentration and incubated for 72h. The mean and SDs of the plots were obtained from 3 wells within 3 independent assays. Asterisk (*) indicates a significant difference. ****P < 0.0001 two-tailed Student's t test.

Figure S2

MLN8237 induced polyploidy of gastric cancer cells. AGS cells were treated with the indicated MLN8237 concentration for 72h, and subjected to DAPI staining and FCM to determine the cell cycle profile. The mean and SDs of the plots representative of three independent experimentsplot. Asterisk (*) indicates a significant difference. **P < 0.01, ***P < 0.001, ****P < 0.0001 two-tailed Student's t test.

Figure S3

MLN8237 treatment induced Bax cleavage and p27 degradation is in SMMC-7901 and HepG2.

(a) AGS cells were cultured at the indicated concentration of MLN8237 for 72h and subjected to immunoblot.

Figure S4

AURKA knockdown enhanced MLN8237-dependent p27 downregulation and Bax cleavage. (a) p27 turnover and Bax cleavage were AURKA-dependent. AGS cells were transfected with negative control of siRNA or p27 siRNA for 24-96h and then subjected to immunoblotting analysis for indicated protein expression. (b) AURKA knockdown enhanced MLN8237-induced p27 degradation and Bax cleavage. AGS Cells were cultured in 200nM MLN8237 after AURKA knockdown and subjected to immunoblotting to determine the expression of the indicated proteins. This immunoblot is representative of three independent experiments. (c-d) Calpain 4 (C4) knockdown attenuated AURKA knockdown-dependent apoptosis. Apoptosis was determined by Annexin V/propidium iodide double staining and FCM in each group. The experiment was performed independently three times.

Figure S5

Bax knockdown attenuated MLN8237 or calpain and MLN8237 treatment-induced apoptosis. (a) Bax knockdown attenuated MLN8237-induced apoptosis. 200nM MLN8237 was added to culture after Bax silencing and detected apoptosis at 72h by FCM. (b) Bax knockdown attenuated calpain and MLN8237 double treatment-induced apoptosis. 200nM MLN8237 was added to culture after Bax siRNA and calpain 1/4 (C1) were transfected into AGS and detected apoptosis at 72h by FCM. (c) Trasnfection effect of Bax siRNA and calpain in AGS. The experiment was performed independently three times.

Figure S6

Skp2 and caspase had no contribution to MLN8237-induced p27 degradation and Bax cleavage. (a) 200nM MLN8237 was added to culture after Skp2 siRNA and cultured for 72h. AGS was subjected to immunoblotting to determine the expression of the indicated proteins. (b) 200nM MLN8237 was added to culture or 72h after 10uM Z-VAD-FMK pre-incubated for 4h. This immunoblot is representative of three independent experiments.

Figure S7

MLN8237 induced Ca^2+^ pathway activation. (a) AGS was treated with MLN8237 for 72h at indicated concentration and loaded with 5 μM Fluo-3/AM for 30 min at 37°C. The labeled cells were analysis by photographed through a fluorescence microscope.

Figure S8

p21 knockdown did not enhance MLN8237 cytotoxicity to AGS gastric cancer cells. (a) p21 knockdown partly reversed cell proliferation inhibition upon 200nM MLN8237 treatment for 72h. AGS proliferation of indicated treatment was measured by MTS assay. Error bars represent SD from three independent experiments. (b) p21 knockdown did not augment 200nM MLN8237-induced Bax cleavage when incubated for 72h. Cells were subjected to immunoblotting to determine the expression of the indicated proteins. This immunoblot is representative of three independent experiments. Asterisk (*) indicates a significant difference. **P < 0.01 two-tailed Student's t test.

Figure S9

p38/MAPK inhibition sensitized cytotoxicity of MLN8237. (a) 200nM MLN8237 treatment for 72h led to p-p38/MAPK downregulation in AGS. Cells were subjected to immunoblotting to determine the expression of the indicated proteins. (b-d) The combination of SB203580 and MLN8237 enhanced AGS cancer cell destruction at 72h. Error bars represent SD from three independent experiments. (e) SB203580 treatment increased MLN8237-induced Bax cleavage. AGS was treated with 25 µM SB203580 for 4 h prior to MLN8237 treatment and incubated for 72h. This immunoblot is representative of three independent experiments. Asterisk (*) indicates a significant difference. ****P < 0.0001 two-tailed Student's t test.

Figure S10

Higher expression of Bax induced apoptosis in 293T and AGS. 293T(a) and AGS(b) was transfected with indicated amount of Bax-HA and detected apoptosis by FCM at 24h. Asterisk (*) indicates a significant difference. ****P < 0.0001 two-tailed Student's t test.
